# A survey of resistance to colchicine treatment for French patients with familial Mediterranean fever

**DOI:** 10.1186/s13023-017-0609-1

**Published:** 2017-03-16

**Authors:** Alice Corsia, Sophie Georgin-Lavialle, Véronique Hentgen, Eric Hachulla, Gilles Grateau, Albert Faye, Pierre Quartier, Linda Rossi-Semerano, Isabelle Koné-Paut

**Affiliations:** 1 Department of Paediatrics Rheumatology, CEREMAI, Bicêtre Hospital, AP-HP, University of Paris SUD, Le Kremlin-Bicêtre, France; 2 Internal medicine, CERAIF, Tenon hospital, AP-HP, University of Paris EsT, Paris, France; 3 Paediatrics, CEREMAI, Mignot Hospital, Versailles-Le Chainay, France; 4 Internal medicine, Huriez hospital, University of Lille, Lille, France; 5 General Paediatrics, Robert Debré Hospital, AP-HP, University of Paris, Paris, France; 6 Paediatric rheumatology and immunology, CERHUMIP, Necker Hospital, University of Paris, Paris, France

**Keywords:** Familial Mediterranean fever, Colchicine, Resistance to treatment, Compliance, Anti interleukin 1

## Abstract

**Background:**

Colchicine is the standard treatment for familial Mediterranean fever (FMF), preventing attacks and inflammatory complications. True resistance is rare and yet not clearly defined. We evaluated physicians’ definition of colchicine resistance and report how they manage it.

**Patients and methods:**

We recruited patients with a clinical diagnosis of FMF, one exon-10 Mediterranean fever (*MEFV*) gene mutation and considered resistant to colchicine, via networks of expert physicians. Clinical, biological characteristics and information about colchicine treatment (dose adjustment, compliance) were collected. The severity of FMF was assessed by the Tel Hashomer criteria.

**Results:**

We included 51 patients, most females (55%), mean age 34 ± 23.1 years years (range 4.7–86.3). Overall, 58% (27/47) patients had homozygous M694 *MEFV* gene mutations. Seventeen of 42 patients (40%) declared full adherence to colchicine treatment, greater for children (48%) than adults (22%). Physicians considered colchicine resistance with > 6 attacks/year (*n* = 21/51, 42%), > 4 attacks in the last 6 months (*n* = 13/51, 26%), persistent inflammation (*n* = 23/51, 45%), renal amyloidosis in (*n* = 6/28, 22%) of adult patients and intolerance to an increase in colchicine dose (*n* = 10/51, 19%), and other reasons (*n* = 13/51, 23%), including chronic arthralgia (*n* = 6/51, 12%). Interleukin 1–targeting drugs represented the only alternative treatments in addition to daily colchicine.

**Conclusion:**

Resistance to colchicine is rare (<10% of patients) and mostly observed in severe *MEFV* genotypes. The main reasons for physicians assessing resistance were severe clinical symptoms, persistent subclinical inflammation, and secondary amyloidosis. Low adherence to colchicine treatment is a key component of resistance.

## Significance and innovations

- In pediatric care setting, the most important reason to consider resistance to colchicine treatment in patients with FMF was a high frequency of attacks.
- In adult care setting, the most important reason to consider resistance to colchicine treatment was secondary amyloidosis.
- In both group, digestive intolerance, persistent subclinical inflammation and joint symptoms contributed to colchicine resistance.
- Overall full compliance to colchicine treatment was low (40%), especially in the adult care setting group (22%)

## Background

Familial Mediterranean fever (FMF) is the historical prototype of a group of inherited inflammatory disorders of innate immunity, so-called autoinflammatory diseases. FMF is essentially observed in Mediterranean populations, affecting more than 100,000 people [1]. The main clinical characteristics are self-limited acute febrile attacks accompanied by peritoneum, pleura, skin, muscle and joint inflammation. FMF severely impairs quality of life and causes secondary inflammatory complications such as amyloid A amyloidosis [2].

Daily treatment with colchicine was introduced in 1972 to prevent FMF attacks and secondary amyloidosis by also reducing the level of sub-clinical inflammation [2]. The action mechanisms of colchicine are diverse and still unclear, but its ability to disrupt the cytoskeleton probably plays an important part. Colchicine may have anti-inflammatory effects in FMF by reorganizing the actin cytoskeleton and down regulating Mediterranean fever (*MEFV*) gene expression. To date, a median dose of 1–2 mg daily of colchicine remains the mainstay of FMF treatment, allowing for significant reduction or absence of acute attacks in more than 90% of cases [3, 4].

Although considered generally safe and effective, daily colchicine treatment for FMF has some limitations. Indeed, colchicine has a narrow therapeutic window at blood levels < 7 ng/mL, but at doses > 10 ng/mL, it has serious toxic effects and can lead to potentially fatal outcomes. This peculiarity is also associated with digestive intolerance, which limits the possibility of increasing the daily dose to obtain full therapeutic effect in patients with the most severe (inflammatory) phenotypes. In daily practice, although colchicine remains an inexpensive and effective means to control FMF inflammation, 5 to 10% of patients will not be able to achieve complete response, which raises the possibility of new therapeutic approaches such as interleukin 1 (IL-1)-targeting drugs [5–7].

Considering the very high cost of these treatments for FMF, we aimed to survey when and how adult and paediatric physicians, consider resistance to colchicine in patients with FMF and to report how they handle this situation in their practice.

## Patients and methods

### Patients and setting

We retrospectively reviewed charts of patients identified through reference centres and networks of inflammatory-disease expert physicians. Electronic mailing lists of French paediatric and adult rheumatologist societies were used to request medical histories of FMF patients considered resistant to colchicine treatment. We asked physicians to report patients with a clinical diagnosis of FMF and at least one pathogenic *MEFV* mutation whom they considered resistant to colchicine. A dedicated questionnaire was used to collect data on demographics (age, sex, ethnic origin), *MEFV* mutation type, age at first symptoms and at diagnosis, description of clinical symptoms before and under colchicine treatment, biological inflammatory markers tested during and between attack periods before and during colchicine treatment, associated inflammatory diseases, tolerance to treatment, dose adjustments, and evaluation of adherence to treatment. Disease severity was assessed by the Tel Hashomer criteria [8]. Finally, we analysed the reasons for physicians considering their patients resistant to treatment and collected the alternative attitudes and treatments used. We excluded patients with concomitant diseases and manifestations that might mimic FMF, such as spondyloarthropathies or Crohn’s disease, to avoid confusion in evaluating disease severity.

### Statistical analysis

Because we had both paediatric and adult care setting populations, we first divided the patients into these two subgroups, with the paediatric care population ranging in age from 0 to 21 years. We chose this age limit because several patients were still seeing paediatricians from age 18 to 21 years. General statistics are reported as mean ± SD. All descriptive results are given with 95% confidence intervals (95% CIs). Analyses involved the chi-square test for categorical variables and *t*-test for continuous variables. Microsoft Excel vXI was used for analysis. *P* < 0.05 was considered statistically significant.

## Results

### Study patients

We recruited 51 patients with a clinical diagnosis of FMF from nine centres, in which four were paediatric departments. Mean age was 34 ± 23.1 years (range 4.7–86.3), with 23 males (45%); 23 patients (45%) under 21 years old were in paediatric care setting, in whom 3 between 18 and 21 years old. Thirty (59%) were Sephardic Jews, seven (14%) were from Turkey or Armenia, ten (20%) were from North Africa, two (4%) were from Lebanon and two (4%) were of mixed ethnic background. The mean age at disease onset was 7.8 ± 8.7 years (range 1 month to 40 years); 17 (33%) had disease onset ≤ 2 years. All patients with available data (*n* = 47/51, 93%) carried pathogenic mutations in exon 10 of *MEFV* except one girl and a woman with a complex allele including *MEFV* deletion. (Table 1).

**Table 1:** Clinical and demographic characteristics of 51 patients with familial Mediterranean fever before colchicine treatment

	All patients *n* = 51	Group I^a^ *n* = 28	Group II^b^ *n* = 23	*p values*
Age at diagnosis (years)	7.8 ± 8.7	12.2 ± 9.6	2.7 ± 2.8	0.001
Sex				0.166
Male	23 (45%)	10 (36%)	13 (57%)	
Female	28 (55%)	18 (64%)	10 (43%)	
*MEFV mutation*				0.235
*M694V/M694V*	27 (51%)	13 (43%)	14 (61%)	
*M694V/M694I*	7 (13%)	4 (13%)	3 (13%)	
*M694I/M694I*	1 (2%)	0 (0%)	1 (4%)	
*M680I/M680I*	1 (2%)	0 (0%)	1 (4%)	
*M694V/V726A*	1 (2%)	1 (3%)	0 (0%)	
*M694V/I591T*	1 (2%)	1 (3%)	0 (0%)	
*M694V/E148Q*	1 (2%)	1 (3%)	0 (0%)	
*M726V/M680V*	1 (2%)	1 (3%)	0 (0%)	
*M694V/TRAPS polymorph.*	1 (2%)	0 (0%)	1 (4%)	
*E148Q/I692del/V726A*	1 (2%)	0 (0%)	1 (4%)	
*M694V/-*	4 (8%)	1 (3%)	3 (13%)	
*M694I/-*	1 (2%)	1 (3%)	0 (0%)	
NA	4 (7.5%)	4 (13%)	0 (0%)	
Frequency of attacks				0.019
> 1/2 weeks	13 (26%)	5 (18%)	8 (35%)	
> 1/month	17 (33%)	7 (25%)	10 (44%)	
> 1/3 months, <1/month	3 (6%)	1 (4%)	2 (9%)	
< 1/3 months, >3/year	2 (4%)	1 (4%)	1 (4%)	
< 3/year	1 (2%)	0 (0%)	1 (4%)	
NA	15 (29%)	14 (50%)	1 (4%)	
Duration of attacks (hr)	57 ± 22.4	67 ± 17	50 ± 24	0.025
NA	17 (33%)	13 (46%)	4 (17%)	
Delay to treatment (years)	8.7 ± 12.9	15 ± 15.9	2.4 ± 2.6	<0.001
Total	51	28 (55%)	23 (45%)	

Data are n (%) or mean ± SD

^a^Group I: Adult care setting

^b^Group II: Pediatric care setting

#### Frequency and duration of attacks before and after colchicine treatment

We had data for 36 patients (72%) before colchicine treatment; 14 (50%) patients in adult care setting (group I), 22 (96%) patients in pediatric care setting (group II), (Table 1). The frequency of attacks was significantly higher in patients of group II than in those of group I (*p =* 0.019). The mean duration of attacks in group II was 50 h (range 12–84), which was significantly lower than in group I: 67 h (range 36–96; *p =* 0.025). Under colchicine treatment, for 27 (53%) patients, the number of attacks was > 1/month (15 (65%) in group II vs. 12 (43%) in group I; *p* = 0.27). These data were unavailable for 28% of patients (4% in group II vs. 47% in group I). The mean duration of attacks under colchicine treatment was 55 h (range 12–120): 50.5 h (range 24–120) in group II vs. 60 h (range 11–96) in group I *(p* = 0.29).

#### Severity of attacks before and during colchicine treatment

We had data before colchicine treatment for 22 (43%) patients for subjective evaluation, because the Tel Hashomer criteria include the response to colchicine. For all patients, the attack severity was > 4/10 and for 13 (59%), it was > 7/10. During colchicine treatment, all 51 patients were classified by disease severity: disease was severe for 41% (*n* = 21), intermediate for 35% (*n* = 18), and mild for 23% (*n* = 12).

#### Clinical features before and during colchicine treatment

Before Colchicine treatment, 44 patients (83%) had fever during attack periods; these data were unavailable for 3 patients in group I (10%). Overall, 33 patients (73%) had abdominal pain: 91% in group II versus 57% in group I (*p* = 0.009). During Colchicine treatment, 10 (22%) of patients had myalgia during attack periods, 2 (8%) in group I vs 8 (36%) in group II, (*p* = 0.21). Overall symptoms during attacks before and under colchicine treatment are detailed in Tables 2 and 3.

**Table 2:** Patient’s clinical symptoms before colchicine therapy

	Population *n* (%)	Group I^a^ *n* (%)	Group II^b^ *n* (%)	*p* values ^¥^
Data available	45 (88%)	22 (79%)	23 (100%)	
Fever	44 (83%)	21 (70%)	23 (100%)	*0.3*
Abdominal pain	33 (73%)	20 (91%)	13 (57%)	*0.009*
Arthralgia	21 (41%)	11 (39%)	10 (44%)	*0.494*
Asthenia	2 (4%)	1 (4%)	1 (5%)	*0.974*
Myalgia	8 (18%)	2 (9%)	6 (26%)	*0.136*
Thoracic pain	10 (22%)	6 (27%)	4 (17%)	*0.4*
Diarrhoea	2 (4%)	1 (5%)	1 (4%)	*0.974*
Amyloidosis	1 (2%)	1 (5%)	0 (0%)	*0.301*
Skin rash	15 (33%)	8 (36%)	7 (30%)	*0.673*
Arthritis	6 (13%)	4 (18%)	2 (9%)	*0.349*
Peritonitis	8 (18%)	2 (9%)	6 (26%)	*0.14*
Vomiting	2 (4%)	1 (5%)	1 (4%)	*0.974*
Headache	3 (7%)	0 (0%)	3 (13%)	*0.08*

^¥^chi square test

^a^Group I: Adult care setting

^b^Group II: Pediatric care setting

**Table 3:** Patient’s clinical symptoms under colchicine therapy

Symptoms	Population *n* (%)	Group I^a^ *n* (%)	Group II^b^ *n* (%)	*p value* ^¥^
Data available	46 (90%)	24 (86%)	22 (96%)	
Fever	40 (87%)	20 (82%)	20 (91%)	0.446
Abdominal pain	37 (80%)	19 (79%)	18 (82%)	0.821
Arthralgia	21 (46%)	11 (46%)	10 (46%)	0.979
Asthenia	13 (28%)	5 (21%)	8 (36%)	0.243
Myalgia	10 (22%)	2 (8%)	8 (36%)	0.021
Thoracic pain	9 (20%)	4 (17%)	5 (23%)	0.605
Diarrhoea	9 (20%)	2 (8%)	7 (32%)	0.045
Amyloidosis	9 (20%)	9 (38%)	0 (0%)	0.001
Skin rash	8 (17%)	2 (8%)	6 (27%)	0.090
Arthritis	7 (15%)	5 (21%)	2 (9%)	0.268
Peritonitis	6 (13%)	3 (13%)	3 (14%)	0.909
Vomiting	5 (11%)	4 (17%)	1 (5%)	0.187
Headache	5 (11%)	1 (4%)	4 (18%)	0.127

^¥^chi square test

^a^Group I: Adult care setting

^b^Group II: Pediatric care setting

### Treatment responses and adherence

The mean delay from the first attacks to colchicine treatment was 8.7 ± 12.9 years (range 0–57): 2.4 ± 2.6 years (range 0–9.8) for group II versus 15 ± 15.9 years (range 0–57) for group I (*p* = 0.001). The mean maximal dose of colchicine given was 2.1 ± 0.7 for group I versus 1.8 ± 0.5 mg (equivalent 0.1 ± 0.01 mg/kg) for group II (*p* = 0.006). In all, 30% of group I patients had a dose > 2 mg, with a maximal dose of 3 mg.

A total of 17 patients (32%) had digestive intolerance [nine of group II (39%) and 14 of group I (27%)], with diarrhoea as a major symptom. One patient had severe muscle toxicity, not related to colchicine toxicity. Adherence to colchicine treatment was accurately evaluated in 42 patients (82%): 73% of group I versus 96% of group II (*p =* 0.03). Overall, 40% of patients (17/42) declared being fully adherent: 48% of group II versus 22% of group I patients *(p* = 0.08). Among these 42 patients, only three (7%) had problem of intolerance to Colchicine.

### Inflammatory markers under colchicine treatment

**Fig. 1:**
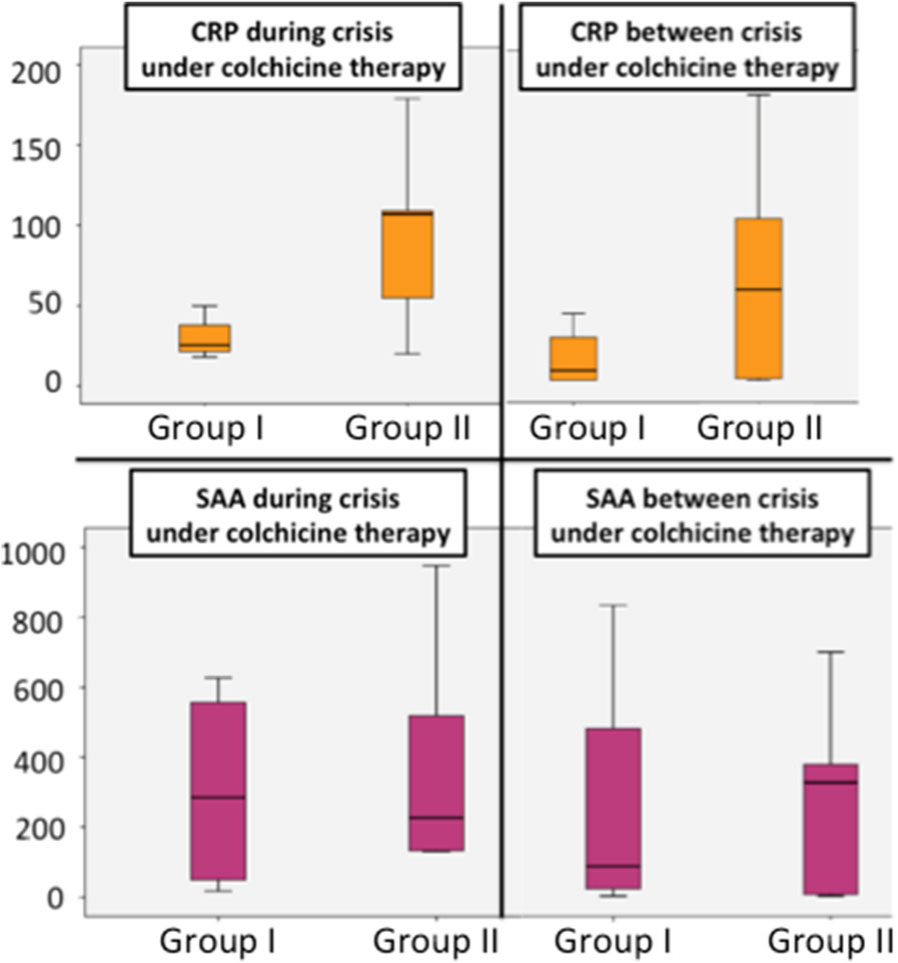
C-reactive protein (CRP) level (mg/L) and serum amyloid A (SAA) (mg/L) levels before and under colchicine treatment. Horizontal lines are means; outer box edges are SD and whiskers are 95% confidence intervals. *Group I: Adult care setting, **Group II: Pediatric care setting

C-reactive protein (CRP) and serum amyloid A protein (SAA) levels during attacks were evaluated in 30 (60%) and 35 patients (69%): mean values were 80 ± 68 and 327 ± 304 mg/L, respectively. Similarly, CRP and SAA levels between attacks were evaluated in 47 (92%) and 35 patients (69%): mean values were 34 ± 44 and 114 ± 202 mg/L, respectively. Levels did not differ between group I and group II populations (Fig. 1). Mean erythrocyte sedimentation rate between attacks was 34 ± 25 mg/L and was available for 28 patients (57%; 83% of group II vs. 32% of group I). Proteinuria between attacks was evaluated in 33 patients (66%), with mean value < 0.1 g/L in group II versus 0.7 g/L in group I, seven patients known to have amyloidosis.

### Determination of inadequate response to colchicine (resistance)

#### Frequency of attacks

Overall, 38% of patients kept a diary of their crises [70% of group II vs 13% of group I (*p < 0.01)*]; 42% were considered resistant to colchicine because of > 6 attacks/year [65% of group II vs 23% of group I (*p =* 0.08)].

#### Amyloidosis and renal failure

In group I, 22% patients were considered resistant because of renal amyloidosis under colchicine treatment (*p = 0.02*), and 20% because of renal failure, which was a contraindication to increase the dose to the optimum *(p = 0.02)* (Table 4)*.*

**Table 4:** Determination of inadequate response to colchicine (resistance)

Symptoms	Population *n* (%)	Group I^a^ *n* (%)	Group II^b^ *n* (%)	*p value* ^¥^
Frequency of attacks				
> 6/year	42%	23%	65%	< 0.08
> 4/6 months	26%	30%	22%	*ns*
Biological inflammation	45%	47%	44%	*ns*
Intolerance to treatment	19%	16%	22%	*ns*
Amyloidosis		22%	0%	*0.02*
Renal failure		20%	0%	*0.02*
Other reasons	23 *%*	18%	26%	*ns*

^¥^By chi-square test

^*^Group I: Adult care setting

^**^Group II: Pediatric care setting

#### Other reasons

In all, 23% of patients had other reasons to be considered resistant to colchicine treatment: 50% had chronic arthralgia (three children <18 years in group II and three patients in group I).

All results are summarized in Table 4

### Concomitant and surrogate treatments

Overall, 60% of patients (70% of group II versus 53% of group I) used concomitant treatments to overcome FMF symptoms: non-steroidal anti-inflammatory drugs (NSAIDs), steroids, or analgesics. Colchicine was still prescribed for 85% of patients, and 62% of these were treated receiving IL-1–targeted drugs (61% in group II vs. 63% in group I). IL-1–targeted drugs were anakinra (79%) or canakinumab (19%), prescribed as continuous treatment for 83% of patients.

## Discussion

To our knowledge, this is the first study evaluating unresponsiveness to colchicine treatment and physicians’ assessment of it in a cohort of both adult and paediatric care patients with FMF living in a western European country. Resistance to colchicine is rare and mostly observed in severe *MEFV* genotypes. Almost two-thirds of our 51 patients had homozygous M694 *MEFV* gene mutations. Less than half of evaluable patients declared full adherence to colchicine treatment, which was greater for children than adults. Physician’s reasons for considering colchicine resistance included > 6 attacks/year, > 4 attacks in the last 6 months, and persistent inflammation. IL-1–targeting drugs represented the only alternative treatments in addition to daily colchicine. The main reasons for assessing resistance were severe clinical symptoms, persistent subclinical inflammation, and secondary amyloidosis. Low adherence to colchicine treatment is a key component of resistance, requiring appropriate patient education.

Renal failure is not a cause for resistance strictly speaking; nevertheless, renal failure impairs the possibility to increase the dose of colchicine. This is why we have considered it as a form of resistance with a cause. The cause of amyloidosis may be multifactorial, and not only related to non adherence or intolerance, but also due to true resistance or additional genetic and environmental factors.

The study was performed within a network of expert tertiary centres, which represents a major strength of the optimal care available in our country. The study gives important data because resistance to colchicine is responsible for increased disease-related morbidity, mortality and poor quality of life with FMF [9, 10]. Thus, resistant patients seem to be good candidates for biologic treatment (i.e., anti-IL-1 treatments). Nevertheless, none of these drugs are being approved, and increasing their use may greatly increase the overall cost of care for FMF [6, 7, 11].

Our study confirmed two important points: first, insufficient response to colchicine treatment is rare (about 10% of all patients seen in our centres); second, insufficient response affects mostly patients with the most severe disease pattern and pathogenic *MEFV* mutations [10]. FMF severity in our patients was reflected by a high number of attacks per year, a high frequency of musculoskeletal involvement, and secondary amyloidosis. Of note, we excluded patients with amyloidosis as a presenting feature of FMF before colchicine treatment. Chronic musculoskeletal symptoms were another cause, well known to be generally benign and overcome with NSAIDs, but in a few cases, they can cause absenteeism from school or work. Some of these patients may show increased risk of developing secondary spondyloarthropathies, which was an exclusion criterion in our study [12]. Secondary amyloidosis appearing during the course of FMF was also a leading cause of resistance to colchicine, exclusively observed in the adult population. Physician’s assessment of resistance to colchicine treatment was in accordance with the definition of the French Israeli consortium, “six or more typical attacks in a year or three in 4–6 months with an elevated acute phase response between attacks,” and with the new EULAR recommendations, at least 1 attack/month in a 6-month period with full adherence to colchicine treatment [4, 13]. Another finding is that a number of patients, especially children, received doses of colchicine higher than that recommended and experienced digestive symptoms of intolerance, which could be considered not strictly synonymous with resistance to treatment. Colchicine is mainly absorbed from jejunal and ileal mucosa and mainly eliminated via biliary excretion (10–20% renal excretion). Anorexia, nausea, diarrhoea, and increased liver enzyme activity are the most common signs of intolerance; these can be overcome in part by twice-daily divided doses, but there are also probably individual differences regarding this issue [14]. Of note, severe fatal intoxications have been reported with concomitant use of clarithromycin and other drugs using the same cytochrome P450 metabolic pathways, which can lead to colchicine accumulation apart from situations of overdose. Alimentary factors may also be involved, and the concomitant intake of grapefruit juice or plants (St. John’s wort) may also increase colchicine toxicity. Another major issue raised by our study and others is the very low full adherence to daily colchicine treatment (40%), even lower than previously reported (60%), which can only be partially explained by digestive intolerance [14]. Indeed, paediatric patients received higher doses than adults and had more side effects but were more fully adherent than adults (48% vs 22%). The international recommendations distinguish low adherence from resistance to colchicine treatment [4, 13], but the practical way to improve the management of this critical issue needs to be determined. New findings have shown that psychological “stress” is sensed by the innate immune system in the brain via the ATP/P2X7R-NLRP3 inflammasome cascade; reversing the activation of this pathway in mice blocked the release of IL-1β (1–3 days after infusion) and produced antidepressant and anxiolytic behavioural effects in non-stressed mice [15]. Many of our patients could benefit from biological treatments that were exclusively anti-IL-1 drugs. Although not within the scope of this study, these treatments gave good response for FMF-related musculoskeletal symptoms and secondary amyloidosis. According to international recommendations, all patients were prescribed daily colchicine, when still possible.

## Conclusion

Despite our retrospective study with some missing data and possible recall bias, it brings important insights into factors affecting inadequate response to colchicine treatment for FMF. Besides the severity of the disease itself, low adherence to treatment is of major importance and remains a challenge for all physicians. Searching for possible causes of digestive intolerance and promoting patient education to reinforce stress control and encourage adherence to treatment may decrease the need for expensive alternative treatments.

## Abbreviations

CRP: C-reactive protein
FMF: Familial mediterranean fever
IL 1: Interleukin 1
*ns*: not significant
NSAIDs: Non-steroidal anti-inflammatory drugs
SAA: Serum amyloid A protein
